# Case of Lithotomy Successfully Performed on a Horse

**Published:** 1824-10

**Authors:** James White

**Affiliations:** Veterinary Surgeon.


ART. X.—*Case of Lithotomy successfully performed on a Horse*. Communicated by JAMES WHITE, Esq. Veterinary Surgeon.

*To the Editors of the London Medical and Physical Journal.*

GENTLEMEN,—The following account of the extraction of a stone from a horse's bladder, has been communicated to me by Mr. W. MOGFORD, who was some years ago my pupil and assistant, and is now a respectable practitioner at North Low, near Okehampton, Devon. Should you think it worth the notice of your readers, I will thank you to insert it in your valuable Journal. I am, Gentlemen, your obedient servant,

JAMES WHITE.

*Wells, Somerset; August 9th*, 1824.

The horse is the property of James Veal, Esq. near Hatherleigh, Devon. When taken up to be broke, he was found very restive, kicking off most of those who attempted to ride him: in consequence of this he received very rough usage, and has since been ridden rather hard. When Mr. Mogford was desired to attend him, he observed a peculiar stiffness in the movement of the hind legs; urine of a high colour and pungent smell, and a dribbling of urine from the penis for some time after staling; pulse between 70 and 80, and hard. By bleeding freely, clysters, fomenting and embrocating the loins, and a week's rest, he appeared sufficiently recovered to be sent to grass. He soon leaped over the gate of the field, and, crossing the country, got back to some pasture where he had been usually kept. This exertion caused a return of his complaint, and Mr. M. was again desired to attend him. He found him in the same state as before described. Wishing to examine the bladder, he introduced his hand into the rectum for that purpose, and immediately felt a hard substance, which appeared to him to be a stone in the bladder. He communicated the circumstance to Mr. Fisher, surgeon, of Hatherleigh, who could not be persuaded that it was a stone, until he had made the examination himself, when he also felt it distinctly. Mr. M. then proceeded to the operation in the following manner:

Having drawn out the penis from the sheath or prepuce, he passed a rod of whalebone up the urethra, until the end of it could be felt in the perineum. He then cut down upon the end of the rod, and through the opening thus made in the urethra he introduced a director, and with a probe-pointed bistoury continued the opening as far as the left side of the anus. He then introduced his right hand into the rectum, and the two fore-fingers of his left hand into the bladder, and, without any difficulty, pushed the stone against the middle finger, by which he guided it to the neck of the bladder, and then easily forced it out through the opening in the urethra. The stone weighed rather more than four and a half ounces. Some parts of the stone appeared to have been broken off and left in the bladder: these were easily removed by means of a bit of soft sponge tied to a whalebone probe, and some warm water. The wound quickly healed, except a small orifice, through which a part of the urine still passes; but the horse has worked hard since, and suffered no inconvenience from it. Mr. M. has no doubt that a stone of seven or eight ounces might be thus extracted.

